# Synthesis of low immunogenicity RNA with high-temperature in vitro transcription

**DOI:** 10.1261/rna.073858.119

**Published:** 2020-03

**Authors:** Monica Z. Wu, Haruichi Asahara, George Tzertzinis, Bijoyita Roy

**Affiliations:** RNA and Genome Editing, New England Biolabs, Inc., Ipswich, Massachusetts 01938, USA

**Keywords:** mRNA synthesis, in vitro transcription, dsRNA by-product, mRNA therapeutics

## Abstract

The use of synthetic RNA for therapeutics requires that the in vitro synthesis process be robust and efficient. The technology used for the synthesis of these in vitro*-*transcribed RNAs, predominantly using phage RNA polymerases (RNAPs), is well established. However, transcripts synthesized with RNAPs are known to display an immune-stimulatory activity in vivo that is often undesirable. Previous studies have identified double-stranded RNA (dsRNA), a major by-product of the in vitro transcription (IVT) process, as a trigger of cellular immune responses. Here we describe the characterization of a high-temperature IVT process using thermostable T7 RNAPs to synthesize functional mRNAs that demonstrate reduced immunogenicity without the need for a post-synthesis purification step. We identify features that drive the production of two kinds of dsRNA by-products—one arising from 3′ extension of the run-off product and one formed by the production of antisense RNAs—and demonstrate that at a high temperature, T7 RNAP has reduced 3′-extension of the run-off product. We show that template-encoded poly(A) tailing does not affect 3′-extension but reduces the formation of the antisense RNA by-products. Combining high-temperature IVT with template-encoded poly(A) tailing prevents the formation of both kinds of dsRNA by-products generating functional mRNAs with reduced immunogenicity.

## INTRODUCTION

One prominent use of in vitro transcription (IVT) in the past few years has been to generate mRNAs for biopharmaceutical and therapeutic applications. The technology used for the synthesis of these in vitro transcribed RNAs—predominantly using phage RNA polymerases (RNAPs)—is robust and well established for the large-scale production of synthetic RNA. However, it is also known that introduction of synthetic in vitro transcribed mRNAs into cells or animal models results in an immune response against the synthetic molecules (Weissman et al. 2000; Karikó et al. 2004; Akira et al. 2006; Sahin et al. 2014; Freund et al. 2019). Such outcomes are undesirable in therapeutic applications in which an immune response is detrimental or unnecessary (for example, protein-replacement therapies). Incorporation of modified nucleosides into synthetic mRNA mitigates the immune response to some extent by mimicking endogenous mRNAs (Karikó et al. 2005; Durbin et al. 2016; Pardi et al. 2017; Richner et al. 2017; Freund et al. 2019). Another major stimulant of the immune response comes from contaminants present in the IVT reactions. A major by-product identified in IVT reactions is double-stranded RNA (dsRNA); this can arise from T7 RNAP's RNA-dependent RNAP activity (Carpousis and Gralla 1980; Martin et al. 1988; Konarska and Sharp 1989; Cazenave and Uhlenbeck 1994; Triana-Alonso et al. 1995; Biebricher and Luce 1996; Arnaud-Barbe et al. 1998). Introduction of transcripts synthesized using T7 RNAP has been shown to activate cytosolic sensors, such as RIG-I and MDA5, that activate the innate immune system in response to viral dsRNA (Kato et al. 2006; Pichlmair et al. 2006; Goubau et al. 2014). Recent studies have identified two main types of by-products in the IVT reaction that result in the formation of dsRNA molecules. The first is formed by 3′-extension of the run-off products annealing to complementary sequences in the body of the run-off transcript either in *cis* (by folding back on the same RNA molecule) or *trans* (annealing to a second RNA molecule) to form extended duplexes (Triana-Alonso et al. 1995; Gholamalipour et al. 2018). The second type of dsRNA molecules is formed by the hybridization of an antisense RNA molecule to the run-off transcript. The antisense RNA molecules have been reported to be formed in a promoter- and run-off transcript-independent manner (Mu et al. 2018). The mechanistic and structural requirements for the formation of these by-products are not well understood, and therefore, it is a challenge to devise methods to prevent their formation or enable their removal.

Because the workflow for therapeutic RNA synthesis has to be compatible with large scale-up processes, preventing the formation of these dsRNA by-products in the reaction is more desirable than adding a post-synthesis purification step, such as chromatography-based purification approaches (Karikó et al. 2011; Weissman et al. 2013; Baiersdörfer et al. 2019). Here we describe a simple and scalable method for preventing the synthesis of dsRNA by-products in the IVT reaction. We describe the use of thermostable T7 RNAPs and the effect of the temperature of the IVT reaction in the formation of dsRNA by-products. We characterize the dsRNA products in the IVT reactions and show that high-temperature transcription results in the reduction of 3′-extended RNA, but not antisense RNA by-products. We also demonstrate that the presence of a template-encoded poly(A) tail has a beneficial effect on the formation of antisense by-products but not on 3′-extended by-products. Finally, we show that mRNAs synthesized with thermostable RNAPs at higher temperatures are functional and have reduced immunogenicity in vivo.

## RESULTS

### 3′-extended by-products are formed during IVT

**FIGURE 1.**
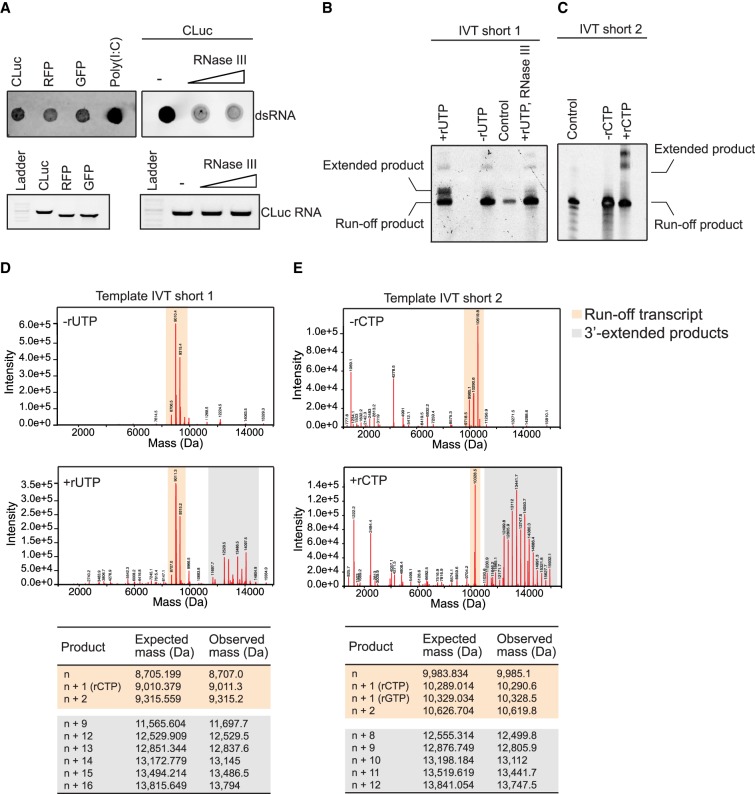
3′-extended RNA by-products are formed during IVT reactions. (*A*) dsRNA immunoblot assay using a dsRNA-specific antibody (J2) on three different mRNA sequences (CLuc, RFP, and GFP). Poly(I:C) and RNase III treatment is used as a control to detect and validate dsRNA, respectively. For the immunoblots, 1.0 µg of RNA was loaded for each mRNA. For the gel electrophoresis analyses, 0.05 µg of RNA was loaded in each well. (*B*,*C*) IVT reactions using short templates (30 bp) run on denaturing gels under standard conditions (four rNTPs), and under conditions where one rNTP was eliminated to prevent formation of the dsRNA products (−rUTP for B and −rCTP for *C*). RNase III digestion control to confirm the dsRNA nature of the extended by-product. Chemically synthesized RNA was used as a control. (*D*,*E*) Intact mass spectrometry of IVT products when all four rNTPs are present (+rUTP for *D* and +rCTP for *E*), and when one rNTP is eliminated to prevent formation of the dsRNA products (−rUTP for *D* and −rCTP for *E*). Expected mass of extended products is calculated based on average rNTP values.

To determine the extent and nature of the dsRNA by-products from IVT reactions, we tested multiple templates of varying lengths and sequences. We used mRNAs encoding for three different proteins (*Cypridina* luciferase [CLuc], a red fluorescent protein [RFP], and green fluorescent protein [GFP]). IVT reactions were performed with wild-type T7 RNAP under standard conditions (5 mM each rNTP; 1 h at 37°C). For detection of the dsRNA by-products, we utilized a standard immunoblot assay that involves recognition of continuous double-stranded structures (>40 bp in length) by a monoclonal antibody (J2) specific for dsRNA (Supplemental Fig. S1; Schonborn et al. 1991; Bonin et al. 2000). Consistent with previous reports, the immunoblot assay showed varying levels of dsRNA by-products in all of the samples, suggesting that under the IVT conditions tested, detectable amounts of dsRNA contaminants are generated (Fig. 1A). Although batch-to-batch variation in the relative amount of dsRNA was detected, overall the trend was consistent. Furthermore, the immunoblot signal diminished when the IVT reactions were treated with RNase III, suggesting that it is generated from bona fide dsRNA contaminants (Fig. 1A). We therefore established that these templates could be used to evaluate the effects of varying reaction conditions and methods to reduce the formation of these dsRNA by-products.

Since the immunoblot assay does not distinguish between the structure or origin of these dsRNA by-products, we established an alternative method for the detection of the dsRNA contaminants. We designed a short DNA template (Template IVT short 1; Supplemental Table S1) that was used to synthesize a 30-nucleotide (nt) run-off RNA product and can be subjected to intact mass spectrometry (MS) analysis. Intact MS analysis would allow for the detection of the run-off product, as well as any other spurious products that are present in the IVT reaction and not resolved by standard gel electrophoresis methods. Furthermore, it could also differentiate between the formation of a dsRNA region due to secondary structures of the run-off RNA from an actual by-product that is different from the run-off transcript. The DNA template sequence was designed with only 3 nt (G, A, and C) so that the IVT reaction could be performed with just three rNTPs and thereby prevent dsRNA formation in the absence of the fourth nucleotide (rUTP) (Supplemental Fig. S2A). The addition of the fourth nucleotide (rUTP) allows base-pairing with adenine in the run-off transcript to form dsRNA regions (Supplemental Fig. S2A). Analysis of the IVT products using denaturing gel electrophoresis showed the presence of the expected run-off transcript compared to a chemically synthesized RNA used as a control when three NTPs (−rUTP lane) were included in the IVT reaction (Fig. 1B). In contrast, two major RNA products were detected when all four NTPs were present in the reaction (Fig. 1B; +rUTP lane). The additional extended RNA product detected, migrated at a higher position suggesting that it is greater in length than the expected run-off transcript (Fig. 1B). Furthermore, RNase III treatment of the products from the IVT reaction with all four NTPs resulted in the disappearance of the higher molecular weight product, confirming its double-stranded nature (Fig. 1B).

The IVT reactions were further subjected to intact MS analysis to identify the molecular weight of all RNA products in the reaction. In the −rUTP reactions, we observed the presence of three main RNA species represented by the expected run-off product (8707 Da), and transcripts that could be attributed to a single nucleotide addition (*n* + 1) to the expected run-off transcript (+C [9011.3 Da]) and some di-nucleotide additions (*n* + 2; +CG [9315.2 Da]) (Fig. 1D). The synthesis of the nontemplated *n* + 1 and *n* + 2 products in IVT reactions has been reported previously and is thought to be an inherent property of T7 RNAP (Milligan et al. 1987; Krupp 1988). In contrast, in the transcription reactions with all four NTPs (+rUTP reactions), in addition to the *n*, *n* + 1, *n* + 2 products, we detected a heterogeneous distribution of RNA products which were up to 22-nt longer (>14,804.9 Da) than the expected 30-nt run-off product (Fig. 1D). RNase III treatment followed by intact mass analysis resulted in the loss of the higher-molecular-weight RNA products but not the *n*, *n* + 1, and *n* + 2 RNA species (Supplemental Fig. S3). In order to rule out potential sequence-specific effects, the same set of experiments was repeated with another template (sequence consisting of G, A, T; Template IVT short 2; Supplemental Fig. S2B), where rCTP could be omitted from the reaction to form the run-off transcript and dsRNA would be formed only when rCTP was added to the reaction (Fig. 1C,E). The same pattern was observed with this second template (Fig. 1C,E). Altogether, the intact mass and denaturing gel analyses of the short IVT templates suggest that during IVT, spurious 3′-extended products are synthesized in the reaction and some of these can form dsRNA structures, supported by their RNase III sensitivity. These observations are consistent with a recent study that demonstrated the synthesis of a heterogeneous population of 3′-extended RNA products during IVT from a completely unrelated DNA template (Gholamalipour et al. 2018).

Taken together, these results demonstrate that intact mass analysis of short template IVT products can be used together with immunoblot analysis of longer mRNAs to evaluate and monitor dsRNA contaminants in IVT reactions.

### Thermostable T7 RNAPs can synthesize RNA efficiently

**FIGURE 2.**
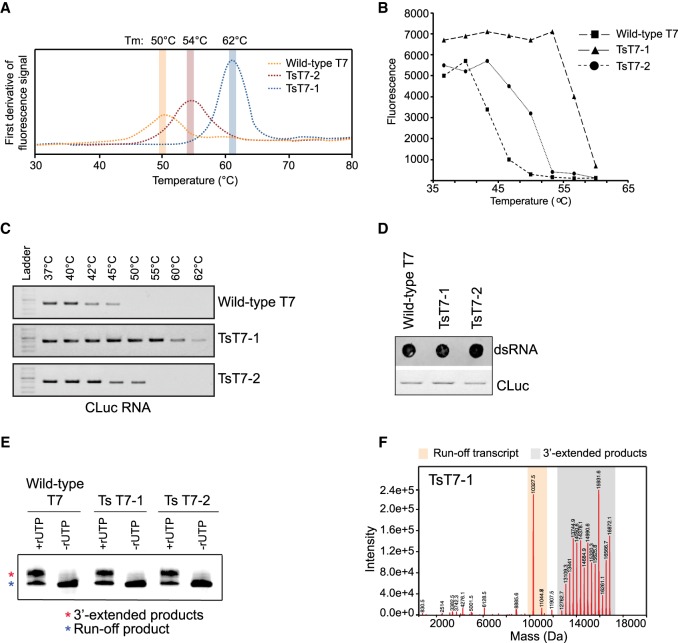
Thermostable (Ts) RNAPs are active at high temperatures and do not affect 3′-extended RNA by-product formation. (*A*) Melting temperatures of wild-type T7 RNAP (orange), TsT7-1 (blue), and TsT7-2 (red) determined by nano-differential scanning fluorimetry. (*B*) Molecular beacon assay for the efficiency of IVT with TsT7-1 or TsT7-2 compared to wild-type RNAP at temperatures ranging from 37°C to 62°C. (*C*) Gel electrophoreses analyses of CLuc RNA synthesized with either wild-type RNAP, TsT7-1, or TsT7-2 RNAPs at temperatures ranging from 37°C to 62°C. Equal volume of IVT reactions were loaded in each well to reflect the differences in RNA yield at each temperature. (*D*) dsRNA immunoblot using J2 antibody and gel electrophoresis analysis of CLuc RNA synthesized from IVT reactions performed at 37°C using wild-type T7, TsT7-1, and TsT7-2 RNAPs. For the immunoblots, 1.0 µg of RNA was loaded for each mRNA. For the gel electrophoresis analyses, 0.05 µg of RNA was loaded in each well. (*E*) Denaturing gel analysis of IVT reactions using Template IVT short-1 with Ts RNAPs compared to wild-type RNAP. Reactions were performed with either four NTPs (+rUTP) or with three NTPs (−rUTP). (*F*) Intact mass spectrometry of short IVT products from TsT7-1 under standard conditions (37°C) with products longer than the run-off transcript highlighted in gray and the expected run-off transcripts in orange (including *n* + 1, *n* + 2 products from nontemplated additions).

Because wild-type T7 RNAP has the propensity to make spurious 3′-extended by-products, we investigated whether changing the reaction conditions could alter the formation of these by-products. One of the proposed mechanisms for the formation of 3′-extended by-products is the rebinding of the run-off transcript by T7 RNAP followed by self-priming (Cazenave and Uhlenbeck 1994; Gholamalipour et al. 2018). We hypothesized that altering the reaction temperature could prevent rebinding of the RNAP to the run-off transcript and thereby reduce the formation of the 3′-extended contaminants. To test this hypothesis, we focused on commercially available thermostable (Ts) RNAPs (TsT7-1 and TsT7-2; Supplemental Fig. S4), which allowed us to raise the temperature of the IVT reaction. As a first step, we evaluated the thermostability of the RNAPs and the optimum temperature for the IVT reactions. As compared to wild-type T7, both TsT7-1 and TsT7-2 had higher thermostability as indicated by the higher melting temperature of the proteins determined using nano-differential scanning fluorimetry (nano-DSF) (Fig. 2A). The TsT7 RNAPs were transcriptionally active at temperatures greater than 45°C, where the activity of wild-type T7 RNAP is compromised, as analyzed with a molecular beacon assay that measures the real-time synthesis of the RNA in the reaction (Fig. 2B) as well as with gel electrophoresis analysis of the CLuc mRNA product (Fig. 2C). Even though TsT7-1 was active at 60°C, high-temperature IVT reactions were performed at 50°C to keep the reaction conditions identical between the two TsT7 polymerases. Importantly, the integrity and yield of the RNAs synthesized from both TsT7 RNAPs were comparable for the templates tested (CLuc; Fig. 2C; Supplemental Fig. S5, Supplemental Table S2).

### Thermostable RNAPs synthesize 3′-extended dsRNA by-products

The two thermostable RNAPs tested in this study have different sets of mutations that confer thermostability to the polymerase (Liao et al. 2003; Sugiyama et al. 2003, 2009; Ong et al. 2017). In order to determine whether the thermostable RNAPs alter the formation of the spurious by-products under standard conditions, IVT reactions were performed under standard conditions (37°C for 1 h) and analyzed by either the dsRNA immunoblot assay (for CLuc mRNA) or by intact mass analysis (for Template IVT short 1). The immunoblot assay showed similar levels of dsRNA contaminants in the reactions with both thermostable RNAPs (Fig. 2D). Additionally, denaturing gel electrophoresis and intact mass analysis of the short IVT RNA showed the presence of the 3′-extended RNA products (Fig. 2E,F), suggesting that the mutations introduced into the thermostable RNAPs do not affect the synthesis of the 3′-extended by-products of the reaction.

### 3′-extended dsRNA by-products are reduced in high-temperature IVT reactions

**FIGURE 3.**
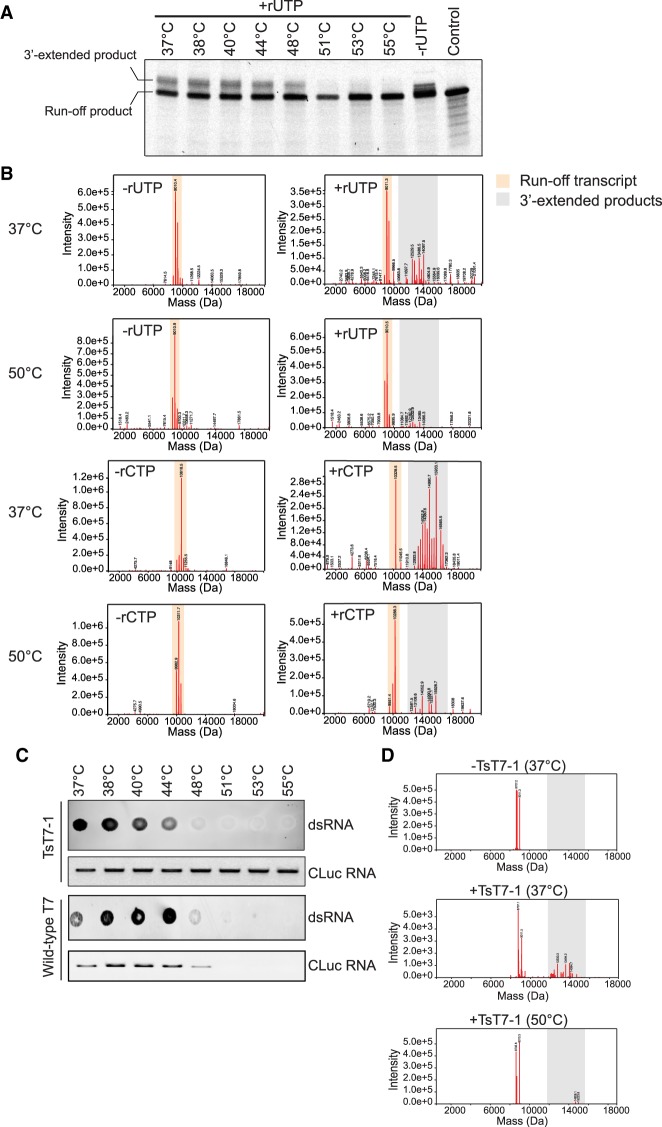
High-temperature IVT with TsT7-1 leads to a reduction in 3′-extended by-products. (*A*) Denaturing gel analysis of IVT products synthesized from short IVT template-1 using TsT7-1 performed at a temperature range of 37°C to 55°C. Chemically synthesized short IVT template 1 RNA was used as a control. Equal amounts were loaded in each lane. (*B*) Intact mass spectrometry analysis of Template IVT short-1 and Template IVT short-2 using TsT7-1 under standard conditions (all four rNTPs; +rUTP or rCTP) or lacking one rNTP (−rUTP or −rCTP) performed at either 37°C or 50°C. Expected run-off transcript (including *n* + 1 and *n* + 2 products) is highlighted in orange, and products longer than the run-off transcript are highlighted in gray. (*C*) dsRNA immunoblot with J2 antibody and gel electrophoresis analysis of CLuc RNA synthesized with TsT7-1 or wild-type RNAP at a temperature range from 37°C to 55°C. For the immunoblots with TsT7-1, 1.0 µg of RNA was loaded for each mRNA. For the gel electrophoresis analyses with TsT7-1, 0.05 µg of RNA was loaded in each well. (*D*) Intact mass spectrometry analysis of IVT reactions in the presence or absence of TsT7-1 on chemically synthesized RNA (*n*, *n* + 1, *n* + 2) performed at either 37°C or 50°C. Products longer than the run-off transcript are highlighted in gray.

We tested the effect of temperature on the formation of the 3′-extended by-products by performing the transcription reactions at temperatures ranging from 37°C to 60°C. Analysis of the short IVT template 1 products by denaturing gel electrophoresis showed the presence of the two RNA products (run-off product and 3′-extended product) in IVT reactions performed at 37°C to 48°C using TsT7-1 (Fig. 3A). However, for transcription reactions that were performed at temperatures >48°C, a single RNA product corresponding to the run-off transcript was observed, suggesting a reduction of the 3′-extended by-products without loss of the run-off product (Fig. 3A). Even though the IVT products were analyzed in a denaturing gel, we wanted to confirm that the absence of the higher-molecular-weight species was not merely due to a change in the conformation of the RNA at the higher temperature. Therefore, we subjected the IVT reactions (from IVT template short 1 and 2) to intact mass analysis. Analyses of the RNA products synthesized at 50°C showed the presence of the run-off transcript and RNA products with the nontemplated additions, but not the 3′-extended products observed in the 37°C reactions (Fig. 3B). Indeed, the reaction products at 50°C were similar to those observed in the −rUTP and −rCTP reactions in Figure 1D,E, respectively. Similar to TsT7-1, IVT reactions performed at 50°C with TsT7-2 also showed reduced amounts of the 3′-extended products (Supplemental Fig. S6). We further tested whether high-temperature transcription could affect the formation of the dsRNAs with templates of different sequences. As expected, when we analyzed the dsRNA contaminants formed in IVT reactions of the CLuc RNA using the dsRNA immunoblot, we observed a reduction in the total dsRNA levels when IVT reactions were performed at 48°C or higher temperatures (Fig. 3C). This is consistent with the reduction in the amounts of the 3′-extended RNA products from the shorter templates and suggests that, even though the RNA immunoblot is detecting a pool of dsRNA contaminants, the majority of them are likely 3′-extended products, whose formation is reduced in high-temperature transcription.

### High-temperature IVT prevents the self-priming of the run-off product

Earlier work has demonstrated that the 3′-extended by-products are synthesized by self-extension of the run-off transcript, leading to the synthesis of considerably longer RNA products during IVT (Cazenave and Uhlenbeck 1994; Triana-Alonso et al. 1995; Gholamalipour et al. 2018). Therefore, we tested whether the 3′-extended by-products we observed from our templates were synthesized by the same mechanism and whether high-temperature IVT prevented the self-extension of the run-off product by T7 RNAP. We chemically synthesized a 30-nt RNA (corresponding to the run-off product from IVT Template short-1), its corresponding *n* + 1, *n* + 2 products, and incubated it in IVT reactions with NTPs (5 mM each) and TsT7-1 RNAP, but in the absence of any DNA template. For reactions that were performed at 37°C, intact MS analysis of the RNA products showed the presence of the 3′-extended products but at reduced levels as compared to when the reaction was done with the DNA template (Fig. 3D). This suggests that in the absence of a promoter sequence, the T7 RNAP can bind an RNA and cause RNA-dependent RNA extension. In contrast, reactions performed at 50°C did not show any detectable 3′-extended products (Fig. 3D). In conclusion, we find that, consistent with previous studies, T7 RNAP can efficiently 3′-extend run-off transcripts (Gholamalipour et al. 2018), and that high-temperature IVT reduces the 3′-extension of the run-off product.

### High-temperature IVT does not affect formation of antisense RNA by-products

**FIGURE 4.**
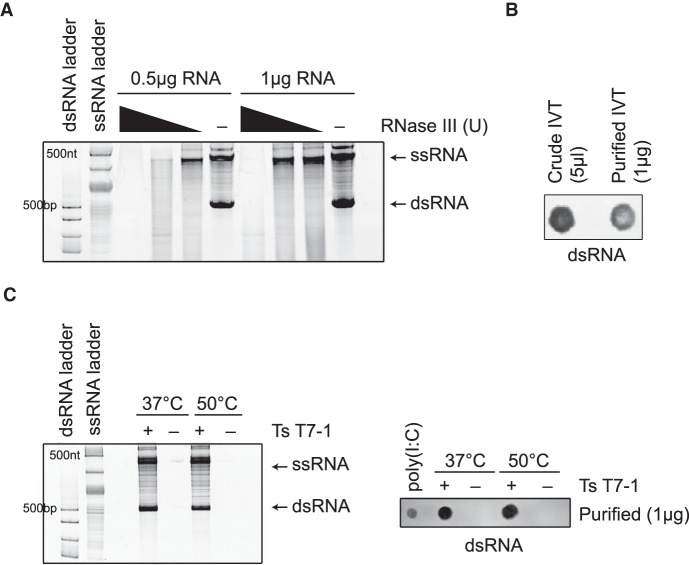
High-temperature IVT does not affect antisense dsRNA by-product formation. (*A*) Native gel electrophoresis analysis of IVT reactions on 512B DNA template using wild-type T7 (37°C) with/without RNase III treatment. (*B*) dsRNA immunoblot with J2 antibody on IVT reactions (crude and purified) with 512B template. (*C*) Native gel electrophoresis analyses and dsRNA immunoblot analysis of 512B IVT reactions conducted with TsT7-1 at 37°C versus 50°C.

A previous report has demonstrated the synthesis of antisense RNA molecules that can base-pair with the run-off transcript and contribute to the formation of dsRNA contaminants in the IVT reaction (Mu et al. 2018). We wanted to investigate whether high-temperature IVT affected the formation of antisense-dependent dsRNA structures. Because we were unable to detect the formation of the antisense RNA in any of our templates under native conditions (data not shown), we tested the same sequence that was reported in the previous study (template 512B) (Mu et al. 2018). With the 512B template, we were able to detect the formation of RNA molecules that were sensitive to RNase III treatment when transcription was performed with either wild-type RNAP (Fig. 4A) or the TsT7-1 RNAP (Fig. 4C). The RNase III-sensitive species were only detected under native gel electrophoresis conditions and not under denaturing gel electrophoresis conditions, suggesting that the run-off transcript and the antisense products had similar migration patterns and were not the same as the 3′-extended products that we and others (Gholamalipour et al. 2018) have observed from other templates. Furthermore, the immunoblot assay was able to detect dsRNA-specific signals from the IVT reactions using the 512B template (Fig. 4B), suggesting that dsRNA species were formed and that the J2 antibody could recognize them irrespective of their nature. To investigate the effect of high-temperature transcription on the formation of the antisense dsRNA species, IVT was performed with TsT7-1 at either 37°C or 50°C. Interestingly, TsT7-1 did not have an effect on the formation of the RNase III-sensitive antisense-mediated dsRNA species at higher temperatures (Fig. 4C). This suggests that the mechanism by which the antisense-mediated dsRNAs are formed is different than that for the 3′-extended dsRNA products, and the conditions that could affect the formation of these are also distinct. Lowering the concentration of magnesium ions in the reaction has been suggested to affect the antisense:sense dsRNA formation (Mu et al. 2018). We tested the effect of lowering the magnesium concentration on the formation of dsRNA by-products. As expected, overall RNA yield was reduced rather than just that of the by-products (data not shown) (Suh et al. 1992; Zaychikov et al. 1997; Kanwal et al. 2018).

### Antisense by-product formation is sequence specific

**FIGURE 5.**
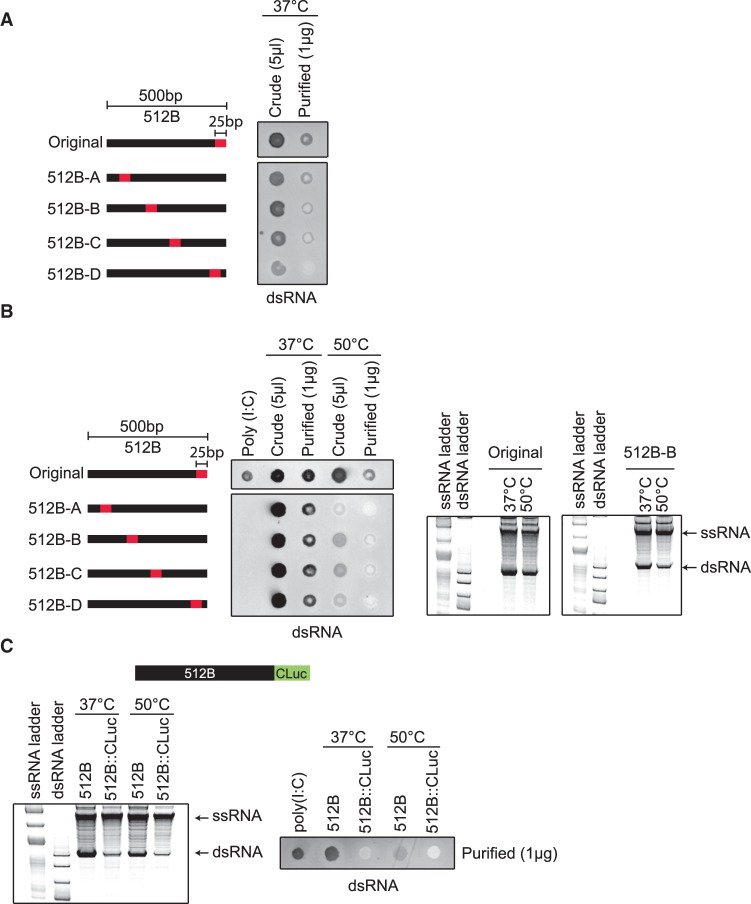
Antisense dsRNA by-product formation is template 3′ sequence dependent. (*A*) dsRNA immunoblot using J2 antibody on IVT reactions with wild-type T7 at 37°C performed on modified 512B templates in which the 3′-terminal 25 bp sequence was moved to various positions within 512B template (512B–A to 512B–D). (*B*) dsRNA immunoblot and native gel analysis of modified 512B templates (512B–A to 512B–D) using TsT7-1 at 37°C versus 50°C. (*C*) A chimeric template was generated in which 26 bp of the CLuc 3′-end sequence was added to the 3′-end of 512B template (denoted 512B::CLuc). Native gel electrophoresis analyses and dsRNA immunoblot analysis of IVT reactions of the 512B::CLuc chimeric template compared to the original unmodified 512B template. Reactions were performed with TsT7-1 at either 37°C or 50°C for 1 h.

The immunoblot assay showed reduction in the dsRNA content when transcription was performed at high temperature for multiple templates (Supplemental Fig. S7). Furthermore, the immunoblot could detect dsRNA irrespective of its source or nature (Figs. 1A, 4B). The fact that high-temperature transcription reduced dsRNA signals from multiple templates (with the exception of the 512B template) suggested that the majority of the dsRNA by-products were 3′-extended and that the antisense by-products from the 512B template might be sequence specific. To understand the formation of the antisense RNA by-products and the effect of high temperature on their synthesis, we constructed chimeric templates in which the 3′-end sequence of the 512B template was altered. Interestingly, moving the terminal 25 base-pair segment away from the 3′ end of the template toward the 5′-end, reduced the level of the antisense dsRNA products from the 512B template when IVT was performed with either wild-type T7 RNAP (Fig. 5A) or TsT7-1 RNAP (Fig. 5B) under standard reaction conditions. We hypothesized that since the presence of such a sequence at the 3′-end of the template increased the propensity of the polymerase to switch to the nontemplate DNA strand and initiate transcription, then adding sequences from the CLuc mRNA 3′-end (512B::CLuc) would alleviate this problem. As expected, when we analyzed the formation of the antisense dsRNA using the 512B::CLuc template, we observed a reduction in the formation of the antisense dsRNA in the reaction (Fig. 5C). Taken together, these data demonstrate that formation of the antisense RNA in the 512B template requires a specific sequence to be present at the 3′-end of the DNA template. Furthermore, it shows that the CLuc RNA 3′ sequence does not have a similar sequence element and, therefore, explains the observed reduction in dsRNA by-products in this mRNA preparation after high-temperature IVT (Fig. 3C).

We next tested the effect of high-temperature transcription on dsRNA by-product formation using the chimeric templates where the extent of antisense RNA product formation has been altered. We observed a reduction in the total amount of dsRNA by immunoblot, as well as a reduction in the dsRNA products from the sense:antisense pairing at higher temperature with TsT7-1 (Fig. 5B). Finally, by truncating the 3′ end of the 512B template by 50 or 200 bp to eliminate the sequence-specific effect of the template on the formation of the antisense RNA by-product, we could detect a reduction in dsRNA levels at higher temperatures (Supplemental Fig. S8).

### Presence of a poly(T) template sequence affects formation of the antisense but not the 3′-extended by-products

**FIGURE 6.**
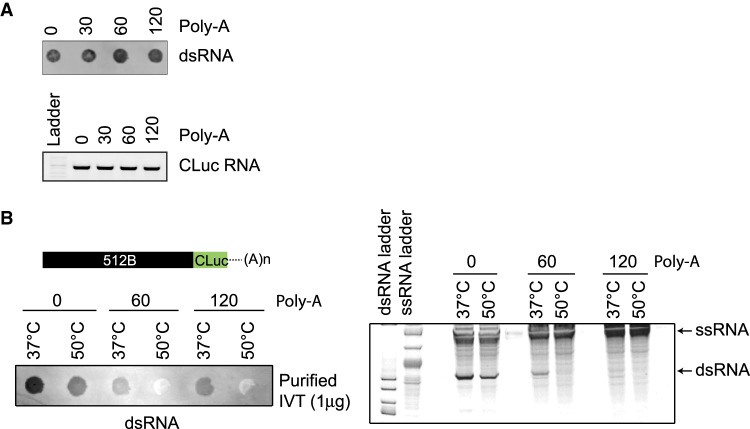
Template-encoded poly(A) tailing reduces antisense by-product formation. (*A*) dsRNA immunoblot with J2 antibody and gel electrophoresis analysis of CLuc RNA synthesized from CLuc templates with varying length (30, 60, 120 bp) of poly(T) sequence at 3′ end under standard conditions. (*B*) Immunoblot and native gel electrophoresis analysis of IVT reactions on 512B::CLuc chimeric template with poly(T) (60 and 120 bp) sequence at the 3′ end. IVT reactions were performed at 37°C or 50°C.

The mechanistic insights on the formation of the two kinds of dsRNA by-products have come from short RNA substrates. However, for the synthesis of functional mRNAs, a poly(A) tail is required, and for most applications, it is encoded from the DNA template. We investigated whether the presence of a poly(A) tail in the 3′ end of the run-off transcript [poly(T) tract in the template DNA] affected the formation of the undesired dsRNA by-products. We generated CLuc DNA templates with varying lengths (30, 60, 120 base pairs) of a poly(T) sequence at the 5′-end, performed IVT reactions under standard conditions (37°C for 1 h), and analyzed the presence of the dsRNA by-products with the immunoblot assay. No substantial differences in the formation of dsRNA by-products was observed when templates with varying lengths of poly(T) were used (Fig. 6A). This observation suggests that at least one, if not both types of the dsRNA by-products were still synthesized when poly(T) sequences are present at the 5′ end of the DNA template.

Based on our data from the 512B-chimeric templates (Fig. 5A–C), we hypothesized that the presence of a poly(T) sequence in the template might be able to override the effect of the antisense-promoting sequence. Varying lengths (60 and 120 bp) of poly(T) sequences were added to the 5′ end of the 512B sequence to test this hypothesis (Fig. 6B). Analyses of the RNA products from the poly(T)-containing 512B templates showed a reduction in the antisense dsRNA species, suggesting that the effect of sequence elements that drive the T7 RNAP toward switching to the nontemplate strand can, in part, be mitigated by the addition of the poly(T) sequences (Fig. 6B). To test whether the addition of the poly(A) tail affected the formation of the 3′-extended dsRNA products, we added poly(T) sequence to the short RNA templates (Template IVT short 1). The synthesis of the 3′-extended products was not affected when poly(T) sequences were present (Supplemental Fig. S9). This suggests that even in the presence of a poly(A) sequence at the 3′-end of the run-off transcript, the RNAP can most likely rebind the run-off product and undergo efficient self-priming.

### Reduced immune response from functional mRNAs synthesized at higher temperatures

**FIGURE 7.**
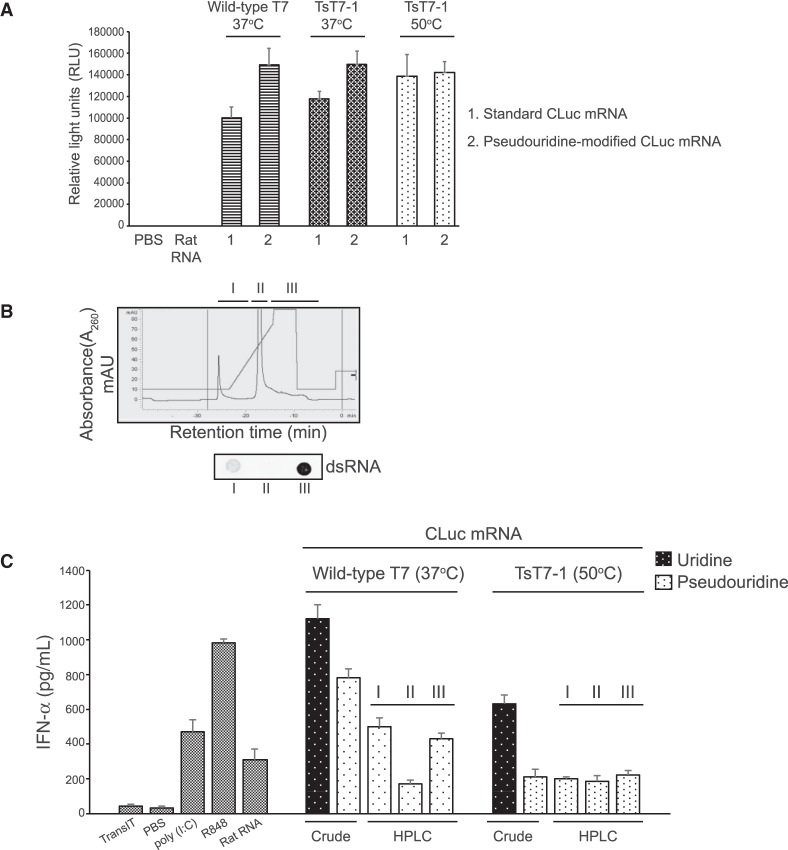
mRNAs generated by high-temperature IVT with TsT7-1 are functional and have reduced immunogenicity in vivo. (*A*) Luciferase activity from HEK293 cells transfected with CLuc mRNA (unmodified or modified with pseudouridine) synthesized with wild-type T7 or TsT7-1 RNAP at 37°C or 50°C. (*B*) Representative chromatogram for CLuc mRNA separated on an HPLC column. Absorbance at 260 nm representing the RNA products in the IVT reaction. dsRNA immunoblot with J2 antibody on fractions collected from the HPLC purification. (*C*) IFN-α levels in supernatant collected from DCs transfected with either crude IVT CLuc mRNA or CLuc mRNA purified with HPLC. IFN-α levels in the supernatant were measured 24 h post-transfection. Error bars are standard error of the mean. Poly(I:C), a synthetic analog of dsRNA and Resiquimod (R848), an activator of Toll-like receptors were used as controls for interferon activation.

Because the presence of dsRNAs in the IVT reaction can stimulate a cellular immune response, we asked whether the reduction in dsRNA by-products observed after high-temperature IVT was sufficient to reduce the immune response observed upon introduction of synthetic mRNAs in cells. We analyzed the immune response from capped and poly(A) tailed CLuc mRNAs synthesized with either wild-type T7 or TsT7-1 RNAPs. Furthermore, to ensure that we are measuring the immune response exclusively from the dsRNA by-products, we introduced pseudouridine modifications in the mRNAs to evade immune response from TLR7 and TLR8 (Karikó et al. 2005, 2008). Comparison of luciferase activity from HEK293 cells that were transfected with CLuc mRNAs synthesized with either wild-type T7 or TsT7 RNAP showed no considerable differences in expression, suggesting that mRNAs synthesized with TsT7-1 at 50°C were functional (Fig. 7A). Comparison of the IFN-α levels from monocyte-derived dendritic cells transfected with CLuc mRNA (transcribed at 37°C or 50°C with wild-type T7 or TsT7-1, respectively) showed that crude IVT CLuc mRNA (without extensive purification) synthesized with wild-type T7 RNAP at 37°C results in secretion of high levels of IFN-α as compared to that seen with control total rat RNA (Fig. 7C). As a comparison for efficient removal of dsRNA by-products from the mRNA preparation, CLuc mRNAs synthesized at 37°C were subjected to high-performance liquid chromatography (HPLC) purification, and fractions either devoid of the dsRNA by-products (Fraction II) or enriched (Fraction I and Fraction III) were transfected. Fraction II resulted in reduced secretion of IFN-α (Fig. 7B). Interestingly, CLuc mRNA synthesized with TsT7-1 at 50°C followed by a silica column cleanup (but no HPLC purification) also showed reduced IFN-α levels as compared with that seen for RNA synthesized at 37°C. The IFN-α levels from the high-temperature IVT mRNAs were comparable to the HPLC-purified Fraction II, suggesting that part of the immune response generated from the presence of the 3′-extended dsRNA by-products in the reaction can be overcome by high-temperature IVT.

## DISCUSSION

It is well established that IVT results in the formation of dsRNA by-products in the reaction, which can have immunostimulatory effects when introduced in vivo (Martin et al. 1988; Triana-Alonso et al. 1995; Karikó et al. 2004). However, the precise nature and source of these by-products is neither well understood nor well characterized. During the course of this work, two independent studies aimed toward understanding the nature of these dsRNA by-products were published (Gholamalipour et al. 2018; Mu et al. 2018). Interestingly, the nature of the dsRNA by-products described in these two studies were very different. The discrepancies between these two studies have not been systematically investigated and reconciled. Furthermore, it is not clear whether the synthesis of the 3′-extended products and the antisense by-products are dependent on each other and whether the parameters that affect the formation of one type of by-product can alter the synthesis of the other type.

Here we demonstrated that high-temperature IVT using thermostable T7 RNAP reduces the formation of dsRNA by-products. Our analyses of the dsRNA by-products using multiple approaches demonstrated that the predominant spurious product generated from the majority of the templates was a 3′-extended species of the main run-off transcript that was able to form RNase III-sensitive dsRNA structures (Fig. 1B–D). This is consistent with observations from the study by Gholamalipour et al. that also identified the 3′-extended products as the predominant type of by-product in IVT reactions and identified the run-off products to be the templates for synthesis of these spurious species (Karikó et al. 2011; Gholamalipour et al. 2018). The mechanism for the formation of these 3′-extended products has been suggested to be the rebinding of the RNAP to the run-off product when the RNA products have accumulated in sufficient amounts in the reaction. The rebinding of the RNAP could essentially result in the folding back of the RNA onto itself followed by extension using the same molecule as the template (self-extension). Our characterization of RNA produced by IVT at higher temperatures demonstrated a reduction in the synthesis of the 3′-extended by-products when the reactions were performed at temperatures greater than 48°C (Fig. 3A–C). It is tempting to speculate that higher temperature alters either the rebinding of the RNAP to the run-off transcript or the folding back and efficient self-priming of the transcript.

In some cases, as exemplified by a specific DNA template in both previous (Mu et al. 2018) and our studies, formation of antisense transcript with a promoter-independent transcription initiation mechanism has been demonstrated where the dsRNA is formed by hybridization of the run-off transcript with the antisense transcript (Mu et al. 2018). Our analyses of this specific DNA template (Fig. 5A–C) demonstrate that the formation of these by-products is sequence specific and requires that sequence to be present at the 3′-end of the template DNA. The exact mechanism by which this sequence induces the transcription of the nontemplate strand by T7 RNAP needs to be investigated.

**FIGURE 8.**
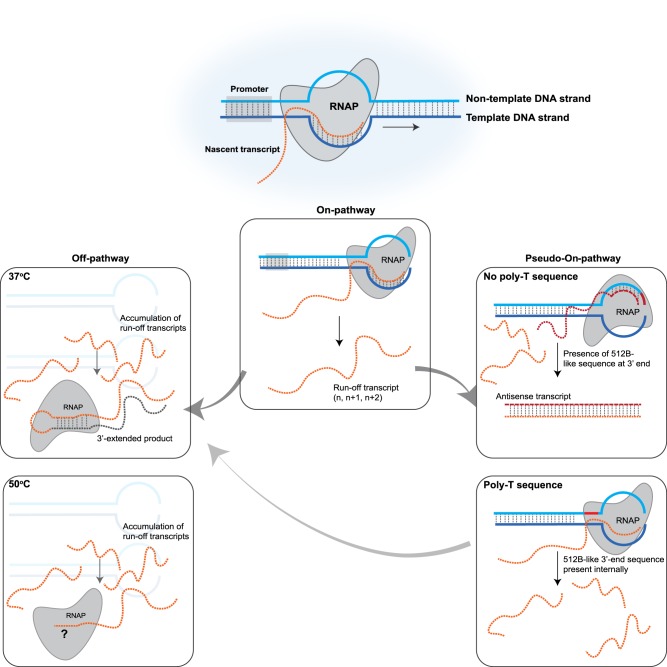
Schematic illustration of competing pathways resulting in the formation of dsRNA by-products during IVT. The canonical “on-pathway” results in promoter-dependent transcription initiation and synthesis of the run-off products (Gholamalipour et al. 2018). Accumulation of the run-off transcripts in the reaction result in a competing “off-pathway” wherein the RNAP rebinds the run-off product and results in self-extension of the run-off transcript. At temperatures higher than 48°C, the 3′-extension of the run-off products are reduced (due to either reduced rebinding of the RNAP to the run-off product or the folding of the run-off product). In the presence of a sequence at the 3′ end of the template (similar to the 512-B 3′-end sequence), the RNAP initiates transcription of the antisense RNA product in a “pseudo-on pathway.” Presence of poly(T) sequences prevent the formation of the antisense RNA products. Presence of a poly(A) sequence at the 3′-end of the run-off transcript does not affect the “off-pathway” at 37°C and can lead to synthesis of longer products if reactions are performed under standard conditions.

Our observations that high-temperature IVT reduced the amount of 3′-extended products, but had little to no effect on the synthesis of the antisense RNA by-product (Fig. 4C) suggest that two competing mechanisms are at play toward the end of the run-off transcription process. In one such mechanism, as suggested by our data (Fig. 5A) and others (Gholamalipour et al. 2018), the polymerase extends the run-off product to form the 3′-extended dsRNA by-products. The self-extension of the run-off transcript is reduced during high-temperature IVT. However, in the presence of a sequence at the 3′ end of the DNA template that promotes the RNAP toward nontemplate strand switching, the RNAP initiates transcription on the nontemplate strand, which results in the formation of the antisense product (Fig. 4A). Altering the reaction temperature has no effect on this process (Fig. 4C). In the presence of a sequence that promotes transcription from the nontemplate DNA strand, 3′-extension might not occur at high enough levels because, instead of rebinding the run-off transcript, the polymerase initiates transcription from the nontemplate strand. The effects of high temperature on the IVT process were apparent in the 512B template when the 3′ DNA template sequence was either moved (Fig. 5C) or substituted with the 3′ sequence of CLuc DNA (Figs. 3C, 5C), which suggests that in the absence of the sequence that promotes synthesis of the antisense RNA by-product, 3′-extended by-product formation becomes more pronounced. This is reinforced by the observation that we could not detect 3′-extended products from the 512B template (data not shown). We hypothesize that rebinding of the RNAP to the run-off transcript might be overridden by the RNAP switching to the nontemplate DNA strand. The synthesis of the 3′-extended product has been shown to be dependent on the accumulation of the run-off transcripts (Gholamalipour et al. 2018). Martin and colleagues have hypothesized that during early IVT, the RNAP binds the promoter and generates run-off products through the canonical “on pathway.” However, when the run-off transcript accumulates to a certain level, the RNAP participates in a competing “off pathway” wherein it rebinds the run-off RNA, which results in self-primed extension. Based on our data, we propose that the presence of a sequence at the 3′ end of the template that allows the RNAP to initiate transcription of the antisense product drives the RNAP to a DNA-sequence-dependent “pseudo-on pathway” that results in the reduction of the 3′-extension (Fig. 8).

Functional mRNAs require a poly(A) tail for stability and efficient protein expression. Poly(A) tails are added to the synthetic RNA either post-transcriptionally with poly(A) polymerase or during transcription via a template-encoded poly(T) sequence. Most previous studies have been conducted on short synthetic RNAs that lack a poly(A) tail; therefore, not much is known about the effects of a poly(T) tract at the 5′ end of the template and its effect on the formation of the spurious by-products. Our data demonstrates that the formation of antisense RNA by-products, which requires a specific sequence at the 3′-end of the transcript, is affected by the presence of the poly(T) sequence (Figs. 5C, 6B). Interestingly, the formation of the 3′-extended products was not affected by the presence of template-encoded poly(A) tails (Fig. 6). Other mechanisms for the synthesis of antisense RNA by-products have recently been proposed (Safran et al. 2019); whether or not high-temperature IVT affects the synthesis of these by-products needs to be investigated.

Recently several different approaches have been proposed to overcome the effects of the dsRNA by-products in vivo. A common approach for removing double-stranded by-products is to use a chromatography-based purification step after completion of the synthesis reaction (Karikó et al. 2011; Weissman et al. 2013). However, implementing such a purification method depending on the nature of the dsRNA by-product, might not be able to remove all contaminants. Selective binding to cellulose has been suggested (Baiersdörfer et al. 2019), but it is not clear whether such a method can distinguish the effects of the intrinsic secondary structures of the RNA. Annealing a DNA oligonucleotide to the 3′ end of the RNA can help overcome the generation of some of the dsRNA by-products (Gholamalipour et al. 2019); for therapeutic applications, however, such a strategy would require specific removal of the DNA oligonucleotide. Finally, lowering the magnesium ion levels in the IVT reaction has been suggested to reduce levels of the dsRNA by-products (Mu et al. 2018), but it also affects the overall yield of the reaction. Furthermore, it is not clear whether the approaches mentioned above will work for the reduction/removal of both kinds of dsRNA by-products that can be made in the reaction.

Our characterization of the thermostable RNAPs (Figs. 2A,B, 7) demonstrate that the RNAs synthesized with the thermostable RNAPs are functional in vivo and demonstrate a reduced immune response in dendritic cells due to reduction in 3′-extended by-products. Furthermore, the combination of template-encoded poly(A) tailing and high-temperature IVT reduces the formation of two types of dsRNA by-products, improves the purity of RNA, and could potentially alleviate the need for extensive post-synthesis purification.

### Summary

Post-synthesis purification strategies have been shown to successfully eliminate the dsRNA by-products from the final mRNA preparations, but the cost associated with them, as well as the incompatibility with scale-up processes, makes them impractical for large scale drug development applications. As outlined in this study, high-temperature IVT with thermostable RNAPs provides a novel, simple, and scalable method to synthesize RNA in vitro*,* especially for applications where generating the run-off products without any other by-product is critical. The generality of this approach for the synthesis of mRNA of any sequence makes it easily adoptable and amenable to be integrated into current RNA synthesis workflows.

## MATERIALS AND METHODS

### Oligonucleotides

All DNA and RNA oligonucleotides for IVT were synthesized by Integrated DNA Technologies (IDT) and the sequences are available in Supplemental Table S1.

### In vitro transcription

For IVT of the shorter oligonucleotides, the forward and reverse oligonucleotides were annealed in DNA annealing buffer (10 mM Tris pH 7.5, 50 mM NaCl, 1 mM EDTA), heated at 95°C for 5 min and then cooled to room temperature. The 512B template was generated with PCR. Transcription reactions were performed with 5 mM of each ribonucleotide triphosphate. Reactions with wild-type T7 RNAP were carried out in 40 mM Tris-HCl pH 7.9, 19 mM MgCl_2_, 5 mM DTT, 1 mM spermidine and supplemented with RNase inhibitor (1000 units/mL) and inorganic pyrophosphatase (4.15 units/mL) (New England Biolabs) at 37°C for 1 h. Reactions with thermostable T7 RNAP (TsT7-1—Hi-T7 RNAP; New England Biolabs and TsT7-2—Thermostable T7 RNAP; Toyobo Life Sciences) were carried out in a buffer containing 40 mM Tris-HCl pH 7.5, 19 mM MgCl_2_, 50 mM NaCl, 5 mM DTT, 1 mM spermidine at varying temperatures (37°C to 60°C) for 1 h. Reactions were supplemented with RNase inhibitor (1000 units/mL) and inorganic pyrophosphatase (4.15 units/mL) (New England Biolabs). The in vitro transcribed RNAs were treated with Turbo DNase (Invitrogen) and purified with the Oligo Clean-Up and Concentration Kit (Norgen Biotek Inc). For the 512B transcripts, reactions were cleaned up with the Monarch RNA Cleanup Kit (New England Biolabs).

IVT of long mRNAs (Cypridina luciferase [CLuc; 1700 nt], red fluorescent protein [RFP; 795 nt] or green fluorescent protein [GFP; 761 nt]) were performed from a linearized plasmid (digested with NotI) or a PCR-amplified linear template. For each reaction, 30 nM DNA template and 30 nM T7 RNAP was used. All four nucleoside triphosphates in the reaction, natural or modified, were used at a final concentration of 4 mM each. For generation of nucleoside-modified mRNAs, UTP was replaced with triphosphate-derivative of pseudouridine (Trilink Biotechnologies). A 120-nt poly(A) tail was template-encoded unless otherwise indicated. The in vitro transcribed RNAs were treated with Turbo DNase (Invitrogen) and followed by spin column clean-up (MEGAclear Transcription Clean-Up Kit). The mRNAs were post-transcriptionally capped with Vaccinia capping enzyme and treated with mRNA Cap 2′-O-Methyltransferase (New England Biolabs).

### T7 RNA polymerase

Wild-type T7 RNAP was purified by Ni-NTA chromatography in 50 mM HEPES KOH pH 7.5, 100 mM NaCl, 10 mM DTT, 0.1% Triton X-100 and stored in 50% glycerol. The thermostable T7 RNAPs (TsT7-1 – Hi-T7 RNA polymerase; New England Biolabs (Ong et al. 2017) and TsT7-2 – Thermostable T7 RNA polymerase; Toyobo Life Sciences (Liao et al. 2003; Sugiyama et al. 2003, 2009) were obtained from the respective manufacturers.

### Molecular beacon assay for in vitro transcription efficiency

Efficiency of IVT reactions was monitored using a modified molecular beacon assay based on a previous study (Marras et al. 2004) and was performed in 40 mM Tris-HCl pH 7.9, 19 mM MgCl_2_, 50 mM NaCl, 5 mM DTT, 1 mM spermidine, 30 nM DNA template, 30 nM T7 RNAP, and 0.5 µM molecular beacon probe. The template was a 22 kb plasmid DNA linearized 6 kb downstream from the T7 promoter, where the beacon target sequence occurs immediately upstream of the linearization site. The target sequence is a 24-nt segment complementary to the loop sequence (underlined) of the DNA oligonucleotide molecular beacon: 5′-CCTGC GATT GAA CAC GTG GGT CAG AGA GG GCAGG-3′. The fluorescent dye 6-FAM was conjugated to the 5′ end and the quencher (BHQ1) to the 3′ end (Integrated DNA Technologies). Reactions were run at various temperatures in the CFX96 Touch Real-Time PCR Detection System (Bio-Rad) for 1 h. End-point fluorescence units for each polymerase were graphed against temperature.

### Melting temperature and thermostability of RNAP

Wild-type T7 RNAP and thermostable T7 RNAPs were diluted to a starting concentration of 2 µg in Tris-HCl, pH 7.5 buffer (40 mM Tris-HCl, 50 mM NaCl, 19 mM MgCl_2_, 1 mM DTT, 2 mM spermidine). Each enzyme was then serially diluted three times at 1:2 ratio for final protein amounts of 2, 1, 0.5, and 0.25 µg. Fluorescence-based thermal unfolding experiments were performed using the Prometheus NT.48 (NanoTemper Technologies). An amount of 5 µL of each sample was loaded into the machine. The temperature was increased by 2°C/min from 20°C to 80°C and the fluorescence at emission wavelengths of 330 and 350 nm was measured. Final data is displayed as the first derivative of the ratio of 350 nm/330 nm emission. Final *T*_m_ for each enzyme is the average of the four dilutions.

### Intact mass analysis of IVT products

IVT reactions were treated with Turbo DNase (Invitrogen) and cleaned up with an Oligo Clean-Up and Concentration Kit (Norgen Biotek Inc). An amount of 100 pmol of each IVT sample was used for intact oligonucleotide mass spectrometry analysis at Novatia, LLC using on-line desalting, flow injection electrospray ionization on a Thermo Fisher Scientific LTQ-XL ion trap mass spectrometer and analyzed with ProMass Deconvolution software.

### dsRNA immunoblot

Crude IVT reactions or purified IVT RNA samples were spotted onto positively charged nylon membranes (Nytran SC, Sigma-Aldrich). The membranes were blocked in 5% (w/v) nonfat dried milk in TBS-T buffer (20 mM Tris, pH 7.4, 150 mM NaCl, 0.1% [v/v] Tween-20). For the detection of dsRNA, the membranes were incubated with J2 anti-dsRNA antibody (1:5000; Scicons) at 4°C overnight. The blots were probed with IR Dye -680 or -800 conjugated secondary antibodies (Cell Signaling Technologies). dsRNA ladder (New England Biolabs) and poly(I:C) (Invivogen) were used as positive controls.

### Gel electrophoresis for antisense and 3′-extended by-product detection

The crude IVT reactions or purified transcripts were analyzed in TBE 20% polyacrylamide gel (native condition) or 6%–15% TBE-Urea polyacrylamide gels (for denaturing condition). Typically, purified IVT RNA was denatured in 2× RNA loading dye (New England Biolabs) and heated at 70°C for 2 min before analyzing on TBE-Urea polyacrylamide gels. SybrGold staining (ThermoFisher Scientific) was performed for RNA visualization and gels were scanned with an Amersham Typhoon Biomolecular Imager (GE Healthcare).

### Cells

Human embryonic kidney cells HEK293 were obtained from American Type Culture Collection and were cultured in Dulbecco's modified Eagle's medium (DMEM) supplemented with l-glutamine (ThermoFisher Scientific) and 10% fetal calf serum. Human dendritic cells were purchased from Lonza Group AG and were differentiated according to the manufacturer's recommendations.

### HPLC purification of IVT mRNA

CLuc mRNA was purified by high-performance liquid chromatography (HPLC) according to a protocol described previously (Karikó et al. 2011) using a linear gradient of 38%–70% buffer B (0.1 M triethylammonium acetate [TEAA], pH 7.0, 25% [v/v] acetonitrile) in buffer A (0.1 M TEAA, pH 7.0) at a flow rate of 5 mL/min. The RNA from the collected fractions was concentrated and desalted with centrifugal filter units (30 kDa MWCO) (Millipore).

### mRNA transfection and expression analyses

Synthesized, purified mRNAs were transfected into human embryonic kidney cells using the TransIT-mRNA Transfection Kit as recommended by the manufacturer (Mirus Bio). The expression of the CLuc mRNA was analyzed by measuring the luciferase activity from the media at various time points (15, 30, 60, 90, 120, 180, 240, 300, 360 min post-transfection). The luciferase activity was measured with BioLux Cypridina Luciferase Assay Kit (New England Biolabs) using a Centro LB 960 luminometer (Berthold) in relative light units (RLU).

### Immune response assay

Dendritic cells (human) were treated with PBS (ThermoFisher Scientific), R-848 (Invivogen), poly(I:C) (Invivogen), or TransIT-complexed IVT RNA. Twenty-four hours post-transfection, the supernatant was harvested and the level of IFN-α was measured as recommended by the manufacturer (PBL Assay Science).

## SUPPLEMENTAL MATERIAL

Supplemental material is available for this article.

## References

[RNA073858WUC1] Akira S, Uematsu S, Takeuchi O. 2006 Pathogen recognition and innate immunity. Cell 124: 783–801. 10.1016/j.cell.2006.02.015 16497588

[RNA073858WUC2] Arnaud-Barbe N, Cheynet-Sauvion V, Oriol G, Mandrand B, Mallet F. 1998 Transcription of RNA templates by T7 RNA polymerase. Nucleic Acids Res 26: 3550–3554. 10.1093/nar/26.15.3550 9671817 PMC147742

[RNA073858WUC3] Baiersdörfer M, Boros G, Muramatsu H, Mahiny A, Vlatkovic I, Sahin U, Karikó K. 2019 A facile method for the removal of dsRNA contaminant from in vitro-transcribed mRNA. Mol Ther Nucleic Acids 15: 26–35. 10.1016/j.omtn.2019.02.018 30933724 PMC6444222

[RNA073858WUC4] Biebricher CK, Luce R. 1996 Template-free generation of RNA species that replicate with bacteriophage T7 RNA polymerase. EMBO J 15: 3458–3465. 10.1002/j.1460-2075.1996.tb00712.x 8670848 PMC451910

[RNA073858WUC5] Bonin M, Oberstrass J, Lukacs N, Ewert K, Oesterschulze E, Kassing R, Nellen W. 2000 Determination of preferential binding sites for anti-dsRNA antibodies on double-stranded RNA by scanning force microscopy. RNA 6: 563–570. 10.1017/S1355838200992318 10786847 PMC1369937

[RNA073858WUC6] Carpousis JA, Gralla JD. 1980 Cycling of ribonucleic acid polymerase to produce oligonucleotides during initiation in vitro at the lac UV5 promoter. Biochemistry 19: 3245–3253. 10.1021/bi00555a023 6996702

[RNA073858WUC7] Cazenave C, Uhlenbeck OC. 1994 RNA template-directed RNA synthesis by T7 RNA polymerase. Proc Natl Acad Sci 91: 6972–6976. 10.1073/pnas.91.15.6972 7518923 PMC44320

[RNA073858WUC8] Durbin AF, Wang C, Marcotrigiano J, Gehrke L. 2016 RNAs containing modified nucleotides fail to trigger RIG-I conformational changes for innate immune signaling. mBio 7 10.1128/mBio.00833-16 PMC5030355 27651356

[RNA073858WUC9] Freund I, Eigenbrod T, Helm M, Dalpke AH. 2019 RNA modifications modulate activation of innate toll-like receptors. Genes (Basel) 10: E92 10.3390/genes10020092. 30699960 PMC6410116

[RNA073858WUC10] Gholamalipour Y, Karunanayake Mudiyanselage A, Martin CT. 2018 3′ end additions by T7 RNA polymerase are RNA self-templated, distributive and diverse in character-RNA-Seq analyses. Nucleic Acids Res 46: 9253–9263. 10.1093/nar/gky796 30219859 PMC6182178

[RNA073858WUC11] Gholamalipour Y, Johnson WC, Martin CT. 2019 Efficient inhibition of RNA self-primed extension by addition of competing 3′-capture DNA-improved RNA synthesis by T7 RNA polymerase. Nucleic Acids Res 47: e118 10.1093/nar/gkz700. 31392994 PMC6821179

[RNA073858WUC12] Goubau D, Schlee M, Deddouche S, Pruijssers AJ, Zillinger T, Goldeck M, Schuberth C, Van der Veen AG, Fujimura T, Rehwinkel J, 2014 Antiviral immunity via RIG-I-mediated recognition of RNA bearing 5′-diphosphates. Nature 514: 372–375. 10.1038/nature13590 25119032 PMC4201573

[RNA073858WUC13] Kanwal F, Chen T, Zhang Y, Zhang Y, Simair A, Rujie C, Sadaf Zaidi NUS, Guo X, Wei X, Siegel G, 2018 Large-scale in vitro transcription, RNA purification and chemical probing analysis. Cell Physiol Biochem 48: 1915–1927. 10.1159/000492512 30092596

[RNA073858WUC14] Karikó K, Ni H, Capodici J, Lamphier M, Weissman D. 2004 mRNA is an endogenous ligand for Toll-like receptor 3. J Biol Chem 279: 12542–12550. 10.1074/jbc.M310175200 14729660

[RNA073858WUC15] Karikó K, Buckstein M, Ni H, Weissman D. 2005 Suppression of RNA recognition by Toll-like receptors: the impact of nucleoside modification and the evolutionary origin of RNA. Immunity 23: 165–175. 10.1016/j.immuni.2005.06.008 16111635

[RNA073858WUC16] Karikó K, Muramatsu H, Welsh FA, Ludwig J, Kato H, Akira S, Weissman D. 2008 Incorporation of pseudouridine into mRNA yields superior nonimmunogenic vector with increased translational capacity and biological stability. Mol Ther 16: 1833–1840. 10.1038/mt.2008.200 18797453 PMC2775451

[RNA073858WUC17] Karikó K, Muramatsu H, Ludwig J, Weissman D. 2011 Generating the optimal mRNA for therapy: HPLC purification eliminates immune activation and improves translation of nucleoside-modified, protein-encoding mRNA. Nucleic Acids Res 39: e142 10.1093/nar/gkr695 21890902 PMC3241667

[RNA073858WUC18] Kato H, Takeuchi O, Sato S, Yoneyama M, Yamamoto M, Matsui K, Uematsu S, Jung A, Kawai T, Ishii KJ, 2006 Differential roles of MDA5 and RIG-I helicases in the recognition of RNA viruses. Nature 441: 101–105. 10.1038/nature04734 16625202

[RNA073858WUC19] Konarska MM, Sharp PA. 1989 Replication of RNA by the DNA-dependent RNA polymerase of phage T7. Cell 57: 423–431. 10.1016/0092-8674(89)90917-3 2720777

[RNA073858WUC20] Krupp G. 1988 RNA synthesis: strategies for the use of bacteriophage RNA polymerase. Gene 72: 75–89. 10.1016/0378-1119(88)90129-1 2468576

[RNA073858WUC21] Liao H, Gemen B, Sugiyama A. 2003 *Mutant of RNA polymerases with increased stability.* U.S. patent no. 6,524,828 B1.

[RNA073858WUC22] Marras SA, Gold B, Kramer FR, Smith I, Tyagi S. 2004 Real-time measurement of in vitro transcription. Nucleic Acids Res 32: e72 10.1093/nar/gnh068 15155820 PMC419623

[RNA073858WUC23] Martin CT, Muller DK, Coleman JE. 1988 Processivity in early stages of transcription by T7 RNA polymerase. Biochemistry 27: 3966–3974. 10.1021/bi00411a012 3415967

[RNA073858WUC24] Milligan JF, Groebe DR, Witherell GW, Uhlenbeck OC. 1987 Oligoribonucleotide synthesis using T7 RNA polymerase and synthetic DNA templates. Nucleic Acids Res 15: 8783–8798. 10.1093/nar/15.21.8783 3684574 PMC306405

[RNA073858WUC25] Mu X, Greenwald E, Ahmad S, Hur S. 2018 An origin of the immunogenicity of in vitro transcribed RNA. Nucleic Acids Res 46: 5239–5249. 10.1093/nar/gky177 29534222 PMC6007322

[RNA073858WUC26] Ong J, Potapov V, Hung K, Asahara H, Tzertzinis G. 2017 *Thermostable variants of T7 RNA polymerase*., U.S. patent no. 0247670 A1.

[RNA073858WUC27] Pardi N, Secreto AJ, Shan X, Debonera F, Glover J, Yi Y, Muramatsu H, Ni H, Mui BL, Tam YK, 2017 Administration of nucleoside-modified mRNA encoding broadly neutralizing antibody protects humanized mice from HIV-1 challenge. Nat Commun 8: 14630 10.1038/ncomms14630 28251988 PMC5337964

[RNA073858WUC28] Pichlmair A, Schulz O, Tan CP, Naslund TI, Liljestrom P, Weber F, Reis e Sousa C. 2006 RIG-I-mediated antiviral responses to single-stranded RNA bearing 5′-phosphates. Science 314: 997–1001. 10.1126/science.1132998 17038589

[RNA073858WUC29] Richner JM, Himansu S, Dowd KA, Butler SL, Salazar V, Fox JM, Julander JG, Tang WW, Shresta S, Pierson TC, 2017 Modified mRNA vaccines protect against zika virus infection. Cell 168: 1114–1125 e1110. 10.1016/j.cell.2017.02.017 28222903 PMC5388441

[RNA073858WUC30] Safran SA, Eckert DM, Leslie EA, Bass BL. 2019 PKR activation by noncanonical ligands: a 5′-triphosphate requirement versus antisense contamination. RNA 25: 1192–1201. 10.1261/rna.071910.119 31239298 PMC6800522

[RNA073858WUC31] Sahin U, Karikó K, Türeci O. 2014 mRNA-based therapeutics–developing a new class of drugs. Nat Rev Drug Discov 13: 759–780. 10.1038/nrd4278 25233993

[RNA073858WUC32] Schonborn J, Oberstrass J, Breyel E, Tittgen J, Schumacher J, Lukacs N. 1991 Monoclonal antibodies to double-stranded RNA as probes of RNA structure in crude nucleic acid extracts. Nucleic Acids Res 19: 2993–3000. 10.1093/nar/19.11.2993 2057357 PMC328262

[RNA073858WUC33] Sugiyama A, Nishiya Y, Kawakami B. 2003 *RNA polymerase mutants with increased thermostability*. U.S. patent no. 0175738 A1.

[RNA073858WUC34] Sugiyama A, Nishiya Y, Kawakami B. 2009 *RNA polymerase mutants with increased thermostability*. U.S. patent no. 7,507,567 B2.

[RNA073858WUC35] Suh WC, Leirmo S, Record MT. 1992 Roles of magnesium in the mechanism of formation and dissociation of open complexes between *Escherichia coli* RNA polymerase and the .lambda.PR promoter: kinetic evidence for a second open complex requiring magnesium. Biochemistry 31: 7815–7825. 10.1021/bi00149a011 1387321

[RNA073858WUC36] Triana-Alonso FJ, Dabrowski M, Wadzack J, Nierhaus KH. 1995 Self-coded 3′-extension of run-off transcripts produces aberrant products during in vitro transcription with T7 RNA polymerase. J Biol Chem 270: 6298–6307. 10.1074/jbc.270.11.6298 7534310

[RNA073858WUC37] Weissman D, Ni H, Scales D, Dude A, Capodici J, McGibney K, Abdool A, Isaacs SN, Cannon G, Karikó K. 2000 HIV gag mRNA transfection of dendritic cells (DC) delivers encoded antigen to MHC class I and II molecules, causes DC maturation, and induces a potent human in vitro primary immune response. J Immunol 165: 4710–4717. 10.4049/jimmunol.165.8.4710 11035115

[RNA073858WUC38] Weissman D, Pardi N, Muramatsu H, Karikó K. 2013 HPLC purification of in vitro transcribed long RNA. Methods Mol Biol 969: 43–54. 10.1007/978-1-62703-260-5_3 23296926

[RNA073858WUC39] Zaychikov E, Denissova L, Meier T, Götte M, Heumann H. 1997 Influence of Mg^2+^ and temperature on formation of the transcription bubble. J Biol Chem 272: 2259–2267. 10.1074/jbc.272.4.2259 8999932

